# Clinical consensus on vestibular infant screening

**DOI:** 10.1007/s00405-025-09833-8

**Published:** 2025-11-23

**Authors:** Jun Yang, Jiali Shen, Yulian Jin, Jianyong Chen, Qing Zhang, Ling Lu, Xiaowan Chen, Hong Yu, Wen Xie, Qingqing Dai, Lian Fang, Yideng Huang, Busheng Tong, Xia Gao, Xiaoyun Qian, Lisheng Yu, Daogong Zhang, Yong Fu, Jie Zhang, Wenyan Li, Pedro Marques, Josine Widdershoven, Raymond van de Berg, Yi-Ho Young, Ilmari Pyykkö, Kimitaka Kaga, Soumit Dasgupta, Maoli Duan

**Affiliations:** 1 Department of Otorhinolaryngology-Head & Neck Surgery, Xinhua Hospital, Shanghai Jiaotong University School of Medicine, Shanghai, China; 2 Shanghai Jiaotong University School of Medicine Ear Institute, Shanghai, China; 3 Shanghai Key Laboratory of Translational Medicine on Ear and Nose Diseases, Shanghai, China; 4 Department of Otolaryngology Head and Neck Surgery, Zhongda Hospital, Southeast University, Nanjing, China; 5 Department of Otolaryngology-Head & Neck Surgery, the First Hospital of Lanzhou University, Lanzhou, Gansu China; 6 Department of Otolaryngology-Head & Neck Surgery, Jilin University, Changchun, Jilin China; 7 Department of Otolaryngology, Head and Neck Surgery, The Second Affiliated Hospital of Nanchang University, Nanchang, Jiangxi China; 8 Department of Otolaryngology, Dizziness Center, West China Hospital, Sichuan University, Chengdu, China; 9 Department of Otolaryngology, The first Affiliated Hospital, Wenzhou Medical University, Zhejiang, China; 10 Department of Otolaryngology Head and Neck Surgery, The first affiliated Hospital, Anhui Medical University, Hefei, China; 11 Department of Otolaryngology-Head & Neck Surgery, Affiliated Drum Tower Hospital of Nanjing University Medical School, Jiangsu Provincial Key Medical Discipline (Laboratory), Nanjing, China; 12 Department of Otolaryngology-Head & Neck Surgery, People’s Hospital of Peking University, Beijing, China; 13 Department of Otolaryngology-Head & Neck Surgery, Provincial Hospital, Shandong University, Jinan, China; 14 Department of Otolaryngology-Head & Neck Surgery, The Children’s Hospital, Zhejiang University School of Medicine, Hangzhou, Zhejiang China; 15 Department of Otolaryngology-Head & Neck Surgery, Beijing Children’s Hospital Affiliated to Capital Medical University, Beijing, China; 16 ENT institute and Otorhinolaryngology Department of Eye & ENT Hospital, State Key Laboratory of Medical Neurobiology and MOE Frontiers Center for Brain Science, Fudan University, Shanghai, China; 17 Unit of Otorhinolaryngology, Department of Surgery & Physiology, University of Porto Medical School, Porto, Portugal; 18 Department of Otorhinolarynology, Maastricht University Medical Center, Maastricht, the Netherlands; 19 Department of Otorhinolarynology, Antwerp University Hospital, Antwerp, Belgium; 20 Department of Otolaryngology, Far Eastern Memorial Hospital, New Taipei, Taiwan China; 21 Hearing and Balance Research Unit, Field of Otolaryngology, School of Medicine, Faculty of Medicine and Health Technology, Tampere University, Tampere, Finland; 22 Department of Otolaryngology and Head and Neck Surgery, Faculty of Medicine, The University of Tokyo, Tokyo, Japan; 23 National Institute of Sensory Organs, National Tokyo Medical Center, Tokyo, Japan; 24 Alder Hey Children’s NHS Foundation Trust, East Prescot Road, Liverpool, L14 5AB UK; 25 Ear Nose and Throat Patient Area, Trauma and Reparative Medicine Theme, Karolinska University Hospital, Stockholm, Sweden; 26 Division of Ear, Nose, and Throat Diseases, Department of Clinical Science, Intervention and Technology, Karolinska Institutet, Stockholm, Sweden

**Keywords:** Cervical vestibular evoked myogenic potentials, Infant, Vestibular infant screening, Sensorineural hearing loss, Vestibular dysfunction

## Abstract

**Purpose:**

To address the current lack of standardized protocols for vestibular infant screening (VIS), this clinical consensus aims to establish a clinical consensus on VIS in infants aged 0–12 months. Given a frequent co-occurrence of vestibular dysfunction in children with sensorineural hearing loss (SNHL) and the significant impact of vestibular dysfunction on early development, early identification and management are critical.

**Methods:**

This clinical consensus was developed by a panel of 28 international experts in pediatric otolaryngology and vestibular disorders through a structured, multi-round Delphi process. Panelists were selected based on clinical and academic expertise, representing 26 institutions across Asia and Europe. A comprehensive literature review (2000–2024) informed the initial draft of consensus statements. The consensus was reached through three iterative rounds of anonymous rating and feedback, followed by virtual discussions. Statements achieving ≥ 80% agreement were retained in the final consensus, which focused on screening tools, target populations, optimal timing, and standardized cVEMP recording protocols for infants.

**Results:**

The expert panel reached consensus on key recommendations in four domains: screening tool, target screening population, timing of vestibular function screening, and recording protocol for cervical vestibular evoked myogenic potential (cVEMP).

**Conclusion:**

This clinical consensus provides foundational guidance for the implementation of VIS in infants, advocating for standardized protocols to improve early diagnosis and intervention. Widespread adoption of these recommendations may enhance developmental outcomes by enabling timely detection and management of vestibular dysfunction in early childhood.

## Introduction

The vestibular system develops in utero, preceding the cochlea, and reaches a well-differentiated state by the 49th day of gestation [1–3]. This process is genetically driven, with fully myelinated vestibular nerves and structured end-organs present at birth [1]. Postnatal maturation, influenced by environmental stimulation, is essential for gross motor function, visual stability, postural control [4–6] and cognition [7]. Vestibular dysfunction can disrupt these processes, leading to delays in motor milestones, impaired gaze stability, and deficits in hand-eye coordination, affecting tasks like reading and writing [8, 9]. It can further impair cognitive functions, resulting in concentration difficulties, memory disturbances, and learning challenges. Psychosocial consequences include fatigue, low self-esteem, withdrawal, anxiety, and avoidance behavior, significantly impacting socio-emotional development [10, 11].

The prevalence of vestibular dysfunction in infants and young children varies between 5% and 25%, depending on study population and method [9]. Conditions that lead to hearing loss, such as genetic mutations, inner ear structural anomalies, inflammatory disorders, trauma, space occupying lesions, ototoxic drugs, noise exposure, and secretory otitis media, can also affect the vestibular system [12–14]. Children with sensorineural hearing loss (SNHL) have a high risk of vestibular dysfunction, with reported prevalence ranging from 20% to 85% [15–18]. Even mild unilateral hearing loss may be accompanied by vestibular dysfunction [4].

Given the common vulnerabilities of the cochlear and vestibular system to various factors and the developmental impact of vestibular dysfunction, early screening and timely intervention are critical [19, 20]. In a pioneering effort, Marten et al. [20] implemented the first vestibular infant screening (VIS) program using bone-conducted vibration cervical vestibular evoked myogenic potentials (BCV-cVEMP) in 6-month-old infants with SNHL in Flanders (Belgium). Their results demonstrated the feasibility of screening, with a 9.5% referral rate in affected infants. Verrecchia et al. [19], the team from Karolinska University Hospital (Sweden), further highlighted the critical role of the vestibular system in infant’s motor development, emphasizing the pressing need for widespread, accessible, safe, and cost-effective early vestibular screening for infants.

Vestibular dysfunction (vertigo) in infants seem to differ from that in children. The latter is often associated with recurrent vertigo of childhood (RVC), vestibular migraines, and otitis media with effusion. Less common causes include Meniere’s disease and vestibular neuritis. Screening revealed that many cases of imbalance (vertigo) in children are underdiagnosed or misattributed to other conditions such as anxiety or behavioral disorders. In infants, vestibular dysfunction may present with nonspecific symptoms such as irritability, vomiting without gastrointestinal illness, poor head control, abnormal head movements, or delays in motor milestones like sitting or crawling.

Despite the recognized morbidity, routine vestibular screening for infants has not been universally adopted, due to several reasons: (1) Firstly, there is a general lack of awareness about the vestibular system, compounded by insufficient specialized training to identify vestibular symptoms and signs, which are often subtle and challenging to detect. Additionally, the limited expressive and communicative abilities of infants and young children further contribute to the frequent oversight of vestibular dysfunction in this age group; (2) The assessment of vestibular function in this population is challenging due to their limited cooperation and the inadequacy of test equipment (such as infant-suitable googles or remote video monitoring system); (3) Lack of standardization in screening tools, target populations and optimal timing; (4) Early screening requires specific equipment, experienced technicians and related pediatric professionals; (5) The absence of established normal reference values across developmental stages; (6) The outcome from vestibular screening is not well established. Unlike hearing loss, where early intervention is well-defined, vestibular deficits may be compensated by cerebral plasticity, rendering some children asymptomatic. The lack of consensus on intervention timing, outcome measurement, and cognitive assessments further complicates the issue.

This consensus document highlights the necessity of VIS, offering implementation guidelines and result interpretation. It synthesizes current literature, clinical practices, and expert recommendations on infant vestibular function and screening.

## Materials and methods

This clinical consensus was developed through a structured, multi-step process involving a panel of 28 international experts in otorhinolaryngology and pediatrics. These participating experts were selected based on their clinical and academic expertise in vestibular disorders, pediatric otology, and vestibular evoked myogenic potentials (VEMPs), with each having a minimum of five years of relevant experience. The panel included representatives from 26 institutions across Asia (China, Japan), Europe (Portugal, the Netherlands, Belgium, Finland, Sweden), and the United Kingdom.

An initial literature review was conducted using PubMed, targeting publications from 2000 to 2024. Keywords included “vestibular infant screening,” “pediatric vestibular disorders,” “cVEMP in infants,” and “sensorineural hearing loss and balance.” Findings from this review informed the first draft of consensus statements.

The consensus-building process spanned March to October 2024 and included three iterative rounds: Round 1: Draft statements were distributed to all panelists. Experts rated their agreement using a 5-point Likert scale (1 = strongly disagree to 5 = strongly agree) and provided open-ended feedback; Round 2: Statements with < 80% agreement were revised based on feedback. The revised statements, along with anonymized comments and aggregated ratings, were redistributed. Panelists re-rated the updated statements; Final Round: Statements with intermediate agreement (70–79%) were further discussed in virtual meetings. Only statements that reached ≥ 80% agreement after discussion were retained in the final version. All experts confirmed formal agreement with the final consensus.

The finalized consensus statements addressed the following four core areas:

- Screening tools appropriate for infants.
- Target screening population.
- Optimal timing for vestibular function screening.
- Standardized recording protocol for cVEMP in infants.

### Recommendation 1: screening tool

Cervical vestibular evoked myogenic potential (cVEMP) as a preferred screening tool to evaluate vestibular function in infants is recommended. Bone-conducted vibration cVEMP (BCV-cVEMP) is more suitable than air-conducted sound cVEMP (ACS-cVEMP).

## Rationale for recommendation 1

Cervical vestibular evoked myogenic potential (cVEMP) is an objective, non-invasive, and well-tolerated test that assesses saccular and inferior vestibular nerve function by measuring myogenic responses in the contracted sternocleidomastoid muscle following high-intensity stimulation. Its feasibility in infants has been well established [21]. Initial studies by Sheykholesami et al. [22], demonstrated the feasibility of ACS-cVEMP in infants aged 1–12 months, noting similarities in overall morphology to adults but with shorter n23 latency and higher amplitude variability in infants. Subsequent investigations by Chen et al. [23] and Erbek et al. [24]. corroborated these findings, indicating that the sacculocollic reflex pathway is responsive at birth and cVEMP testing is viable in newborns, which indirectly proved the well-differentiated saccular structure and complete vestibular nerve myelination. Moreover, studies by Verrecchia et al. [19] and Dhondt et al. [21]. have highlighted the advantages of BCV-cVEMP, such as its high repeatability and success rate in infants, particularly advantageous for those with middle ear pathology which is prevalent in this age group. The response rate of ACS-cVEMP would be influenced by the conductive hearing loss due to attenuation of ACS to the inner ear, while BCV can bypass the middle ear, making it a more suitable option for subjects with middle ear pathology [25]. However, it is important to emphasize that the vibration stimuli are generated by an oscillator, rather than by a conventional vibrator or minishaker. If a vibrator weighing 1.0 kg were to be accidentally dropped during the cVEMP examination, it could cause serious harm to infants.

Video head impulse test (vHIT) objectively assesses semicircular canal function but is limited in infants due to fixation challenges, developmental maturation of the vestibulo-ocular reflex (VOR), and the unavailability of appropriately sized goggles (12). Therefore, an abnormal vHIT result in an infant could be due to either developmental immaturity or a pathological condition, limiting its clinical applicability in this age group [26].

The rotary chair test (RCT) is limited by the potential for skewed results when testing infants on their parent’s lap which could alter the center of rotation and affect gain values. Infants’ variable attention and gaze stability add to the challenge, demanding creative strategies to keep them engaged. Common obstacles to successful RCT include agitation, difficulty in immobilization, premature electrode disconnection and excessive head movement [27].

Given these considerations, BCV-cVEMP emerges as a more suitable option for initial VIS. As a child grows older and the vestibular system develops, more assessment tools, including vHIT, RCT, ocular VEMP (oVEMP), videonystagmography (VNG) to quantify frequency specific vestibular response and caloric test, can be integrated for a comprehensive assessment for vestibular function, as needed.

Notably, cVEMP has predictive value for motor balance in children with hearing impairment. Abnormal cVEMP results correlate with reduced motor capacity, reinforcing its role in early vestibular dysfunction detection and motor delay assessment [28]. Another advantage is its widespread availability, as most auditory brainstem response (ABR) devices include a cVEMP module, facilitating seamless integration into audiology diagnostics. Given its feasibility, high success rate, and practical implementation, cVEMP—particularly BCV-cVEMP, is recommended as the primary tool for early VIS. However, it should be noted that cVEMP cannot assess semicircular canal function, so relying solely on cVEMP for screening may result in some infants and children being missed. Therefore, in cases of strong clinical suspicion or the presence of inner ear abnormalities, even if cVEMP results are normal, additional semicircular canal tests should be conducted whenever feasible.

Importantly, we emphasize that vestibular screening is not equivalent to diagnosis. Instead, we advocate for a stepwise approach, wherein cVEMP serve as a feasible, infant-friendly, and accessible first-line screening tool, especially in settings lacking equipment or personnel for more complex vestibular testing. In cases of abnormal screening results or strong clinical suspicion, comprehensive vestibular evaluation, including semicircular canal function tests such as vHIT and RCT, should follow depends on age and cooperation.

## Recommendation 2: target screening population

Early vestibular screening is strongly recommended for all infants with SNHL. The high risk group includes infants with infections (e.g., congenital cytomegalovirus [cCMV]), cochleovestibular anatomical abnormalities, developmental abnormalities (including genetic syndromes associated with cochleovestibular deficits, gross motor delays, abnormal primitive reflexes, and developmental coordination disorders), as well as infants pre- and post-cochlear implantation.

Motor development varies significantly from one child to another, and some may reach these milestones earlier or later than others. It’s important to consider the range of typical development rather than focusing on exact ages. If there are concerns about a baby’s motor development, consulting a pediatrician or a child development specialist is recommended.

## Rationale for recommendation 2

Evidence across various studies underscore the considerable prevalence of vestibular dysfunction in infants with SNHL. Specifically, Martens et al. [20] revealed a 9.5% referral rate for vestibular dysfunction via BCV-cVEMP in 6-month-old infants with SNHL, emphasizing the profound impact of SNHL on vestibular function. Moreover, Cushing et al. [29] observed a 38% absence rate of ACS-cVEMP in SNHL children, while Verrecchia et al. [19] reported a comparable absence rate of 31.4% with BCV-cVEMP, highlighting the considerable occurrence of concomitant vestibular dysfunction in this group. Additionally, Verbecque et al. [30] identified a significantly higher incidence of vestibular dysfunction in children with SNHL compared with those with normal hearing, reinforcing the critical need for early hearing screening and intervention. As with hearing loss, early intervention for vestibular deficits is expected to yield favorable outcomes.

The severity of SNHL is strongly associated with an increased risk of vestibular dysfunction. Martens et al. [31] identified bilateral severe to profound SNHL as a significant risk factor for abnormal VIS outcomes, with approximately one-third of such infants showing no cVEMP response on the side of hearing loss. Other studies have consistently found a higher prevalence of vestibular abnormalities with worsening SNHL severity [19, 30, 32]. Tribukait et al. [33] observed a marked increase in vestibular dysfunction occurrences when hearing loss exceeded 90 dB nHL, suggesting that profound SNHL often reflects extensive damage to both auditory and vestibular structures. This close anatomical and functional link highlights the necessity for comprehensive assessment and management of both auditory and vestibular function in children with severe SNHL.

Furthermore, certain causes of hearing loss, including cCMV infection, inner ear anomalies, and many genetic conditions ((e.g., Usher, CHARGE, Waardenburg, BOR, Jervell and Lange-Nielsen, DFNB, DFNX, and Pendred syndromes), are strongly associated with vestibular impairment [31, 34], emphasizing the complexity of inner ear disorders and the need for a comprehensive evaluation [18]. A systematic review reported vestibular dysfunction prevalence rates in children with cCMV infection ranging from 14% to 90.4% [35]. Ioue et al. [12] found a higher incidence of vestibular dysfunction in children with cCMV and inner ear malformations compared to those with hereditary non-syndromic hearing loss, emphasizing the importance of detailed vestibular evaluation and ongoing monitoring in this population. However, further research is needed due to the limitation of existing studies, including small sample sizes and inadequate classification of syndromic/non-syndromic hearing loss.

Cochlear implantation is one of the most effective rehabilitation/treatments for patients with severe to profound SNHL, but the procedure carries a risk of damaging the vestibular end organ due to the close anatomical relationship between the cochlea and vestibule. Vestibular dysfunction before and after Cochlear implantation has been reported in up to 50% of patients [36]. Postoperative vestibular deficits may increase the risk of falls, head injuries, and implant damage. Mechanisms include direct trauma from electrode insertion, acute serous labyrinthitis, and vascular or endolymphatic injury due to hemorrhage or infection [36, 37].

Vestibular screening should extend beyond infants with hearing loss to those with motor developmental delays or deficits in age-appropriate fine motor skills. Primitive reflexes—such as the Moro reflex, tonic neck reflex, head-righting reflex, parachute reaction, and Doll’s eye response—are significantly influenced by the vestibular system [38]. These reflexes, persisting up to 12 months, serve as valuable indicators of vestibular function when combined with a comprehensive pediatric motor assessment. Screening should be performed in children presenting with motor deficits, regardless of hearing status.

Another important consideration is making every effort to find out an etiology for SNHL within 3 months following birth and before the vestibular screening. This is a complex medical process and requires dedicated training and experience. The advantages of establishing a cause for SNHL are numerous in terms of parent counselling, prognosticating the natural history of the condition and understanding chances of vestibular involvement.

Therefore, screening the target population from the vestibular point of view is based on a multidisciplinary approach involving different specialists just like newborn hearing screening. It is not limited to only objectively screening a vulnerable population but also addressing children as a whole and their parents.

## Recommendation 3: timing of vestibular function screening

The initiation of vestibular function screening at 3 months of age is recommended. For infants who exhibit abnormal results on their initial screening and present with clear motor developmental delays, abnormal primitive reflexes, or lack of fine motor coordination, immediate intervention, such as vestibular rehabilitation, should be considered. These interventions are crucial to reinforce compensation and support development. For infants who fail the initial screening but do not show the developmental concerns, a follow-up screening at 6 months is advised to monitor for any emerging issues, with subsequent intervention decisions based on the results at that time.

### Rationale for recommendation 3

The 2018 VIS program in Flanders recommended the first vestibular screening at 6 months of age, considering it an ideal time when most infants have developed sufficient head control and sternocleidomastoid muscle level to effectively elicit a cVEMP response [20].

The research conducted by the team in Xinhua hospital (Shanghai, China) demonstrated the feasibility of conducting VIS as early as 3 months [5]. They achieved a 100% success rate in test administration, including Electromyography (EMG) activity and average sweeps in infants of this age group. High response rate for ACS-cVEMP and BCV-cVEMP in 3-month-old infants with normal hearing highlight the sufficiency of neck muscle strength for head support, affirming the practicality of early VIS. In the study, they also found a higher incidence of vestibular dysfunction in infants with SNHL compared with those with normal hearing, suggesting an intrinsic link between hearing loss and vestibular impairments. Therefore, early VIS facilitates the timely detection of vestibular abnormalities and initiation of early interventions, mitigating potential developmental impacts.

According to the Joint Committee on Infant Hearing’s guidelines [39], it is highly recommended that infants with congenital hearing loss undergo early detection within the first month of life. A diagnosis should follow by 3 months, with intervention ideally beginning by 6 months. Conducting VIS at 3 months aligns with this early assessment principle and provides valuable insight into the infant’s inner ear vestibular function. For infants who exhibit abnormal screening results at this stage, a follow-up screening at 6 months is advised. This coincides with the second diagnostic hearing assessment and possible hearing aid fitting [39–41], streamlining the process and improving the likelihood of high participation rates by minimizing the number of hospital visits. This approach also ensures a more comprehensive evaluation and management of both hearing and vestibular health in infants.

Early screening at 3 months may not be universally achievable at this time due to equipment and personnel constraints. Nonetheless, it represents a viable target for tertiary centers or specialized clinics. A flexible, staged implementation, adapted to local resources, is encouraged.

## Recommendation 4: recording protocol for cVEMP

Drawing from established guidelines and consensus on clinical testing techniques for cVEMP [5, 19, 42, 43], manufacturers’ directives for equipment operation, substantial clinical experience, and experience in testing infants. The procedure in infants is not the same as in young children or indeed in adults. There are published norms of test values in infants, but it is recommended that centres develop their own norms. The protocol for conducting cVEMP in infants is detailed as follows.

##  Stimulus parameters

Both click and tone burst are effective stimuli, with a preference for a 500 Hz tone burst which is commonly recommended. Stimulus should follow a 1–2−1 pattern: 1 ms rise time, 2 ms platform time and 1 ms fall time, totaling 4 ms in duration. A minimum of 100 sweeps should be averaged, with the procedure repeated at least twice to ensure waveform repeatability. The recording window set from − 20 ms to 80 ms, employing a stimulus rate of 5.1 Hz. The recommended filter settings are a high-pass cutoff between 5 and 30 Hz and a low-pass cutoff ranging from 1000 to 3000 Hz [42].

Due to smaller ear canal volume (ECV) in infants, which may impact the sound pressure level reaching the cochlea, adjustments in stimulus intensity and duration are critical to ensure safe exposure [44]. For ear canal volume ≤ 0.8 ml, the intensity is set at 120 dB SPL, and for volume >0.8 ml, 125 dB SPL is used [45]. BCV-cVEMP parameters are align with those for ACS-cVEMP. The optimal intensity for BCV-cVEMP considers the output capabilities of the stimulator and subject comfort. For infants, the intensity for BCV-cVEMP is typically set at 129.5 dB force level (equal to 60 dB nHL after calibration), with the bone oscillator alternated between the right and left mastoids for individual ear testing [4, 5, 20].

### cVEMP testing procedure

The skin of the electrode site is prepared with 75% alcohol and scrub gently before placing the electrode. Active electrodes are positioned at the upper third of the bilateral sternocleidomastoid muscle, a reference electrode on the suprasternal notch, and a ground electrode on the forehead for both tests. The electrode impedance must be less than 5 kΩ and the impedance between the electrodes is approximately equal [4, 5, 20, 42].

Infants were entirely awake and placed in a supine position on the bed during testing. One audiologist manages the software, while another gently rotates the infant’s head to the opposite side to ensure the sternocleidomastoid muscle is fully contracted, aiming to have the chin touch the shoulder as close as possible. A family member or caregiver assists by comforting the infant and applying gentle pressure to the infant’s shoulder to prevent lifting. Toys and videos can be used to distract the infant. A minimum of two trials per side is necessary to ensure waveform repeatability and accuracy of the test results.

Regardless of whether ACS-cVEMP or BCV-cVEMP is performed, tympanogram must be included to assess middle ear function. Otitis media with effusion (OME) can lead to an absent or diminished ACS-cVEMP response. Therefore, tympanogram can help avoid misinterpretation of ACS-cVEMP results. For BCV-cVEMP, a larger amplitude response may be observed in cases of OME or conditions such as enlarged vestibular aqueduct (EVA), where mixed or conductive hearing loss is present. Tympanogram results can differentiate between these conditions: an abnormal tympanogram typically indicates OME, while EVA usually shows a normal tympanogram.

### Amplitude correction

Since cVEMP amplitude is strongly related to the strength of sternocleidomastoid muscle contraction, the interaural asymmetry ratio (IAR) was calculated using the corrected amplitude to compensate the bilateral amplitude difference caused by uneven EMG activity [42].

The EMG activity is monitored on the screen, and the root-mean-square (RMS) of EMG is calculated using a pre-stimulus window, which is then rectified and averaged to estimate the pre-stimulus tonic EMG activity. The raw amplitude is divided by this mean rectified EMG to calculate the corrected amplitude. The EMG activity is maintained at least>50 µV [4, 5, 20, 42].

### Investigational parameters of cVEMP

Each cVEMP waveform, latencies (p13 latency for the first peak and n23 latency for the second peak), amplitude (p13 peak to n23 peak amplitude) are recorded, IAR is calculated and recorded. IAR = (AL-AS)/(AL + AS) ×100%, where AL represents the larger corrected amplitude, AS is the smaller corrected amplitude [4, 5, 20, 42].

These recommendations are crafted to facilitate precise, safe, and effective cVEMP recordings in infants, acknowledging the specific anatomical and physiological traits of this demographic.

### Recommendation 5: interpretation of cVEMP results

The primary parameters for interpreting cVEMP results include response rate, latency, amplitude and IAR. These parameters are known to be influenced by various factors, including age, EMG activity, testing parameters and posture, as well as the equipment utilized, etc [42]. To enhance the clinical practice of cVEMP test, we suggest that each center establish its own laboratory’s normative values for different age groups, ensuring a more standardized approach to data interpretation.

### Response rate

The absence of a cVEMP can indicate the pathway of the saccule and inferior vestibular nerve involvement. However, the response rate can be influenced by multiple factors: (1) posture: testing infants in a supine position, with assistance from an examiner for head lift and neck rotation, while parents or caregivers ensure shoulder stability, may be helpful for waveform elicitation [5]; (2) Earlier studies primarily utilized the B-71 bone oscillator [9, 19]. However, the B-81 model, known for its higher output power and lower distortion rate, is better suited for generating effective low-frequency energy crucial for VEMP induction [11]; (3) examiner expertise: the experience and skill of the operator can impact cVEMP test results as well. Experienced professionals can adeptly manage testing conditions, including precise placement of earphones or bone vibrators, and ensuring optimal subject posture, thus improving the response rate.

It was indicated that BCV-cVEMP response rate is higher compared with ACS-cVEMP in infants [5]. This could be attributed to the following factors: (1) Conduction pathways: ACS is transmitted via the external canal and middle ear, where condition like cerumen is common in infants, potentially affecting air-conducted stimulus transmission. BCV directly stimulates vestibular organs through bone vibration, avoiding conductive factors in the external canal and middle ear [5, 25]. (2) In young infants, the use of headphones may lead to ear canal collapse, further affecting the transmission of air-conducted stimulus [25, 46]; (3) The stimulation mechanism differ between ACS and BCV. BCV may stimulate a larger number of nerve fibers due to linear skull acceleration, whereas ACS induces stapes vibration to move the endolymph [47]. It is also hypothesized that BCV produces a more effective shearing motion on the otolith membrane, activating more hair cells in the otolith organ compared to ACS [47, 48]. However, the exact mechanism remains to be further studied.

### Latency

Numerous studies have documented age-related trends in cVEMP latency. Children aged 5–17 years display shorter p13 and n23 latencies than that in subject aged 19–40 years group [49]. This may be attributed to shorter neck lengths in younger children, resulting shorter reflex pathways [50]. Neck length has been proposed as an indirect measure of reflex pathway length, with studies showing a positive relationship between neck length and cVEMP latency for necks shorter than 15.3 cm [51, 52]. Latency can also be affected by several factors including the degree of myelination, efficiency of synaptic transmission, length of the reflex pathway, inclination of nerve fibers and conduction speed [17, 27, 53]. With age, the maturation of the reflex pathway and an increase in conduction speed may counterbalance the effect of increased pathway length, causing latency values to align more closely with those of adults [17, 21, 27]. The variability in reported data could stem from difference in age range of populations studied, testing protocol, recording technique and parameter setting for detection, highlighting the necessity for standardized normative values across different age groups.

### Amplitude

Several studies have found a linear relationship between cVEMP amplitude and EMG activity, emphasizing the importance of controlling sternocleidomastoid muscle contraction level for reliable amplitude measurements [11, 18]. Uncontrolled bilateral sternocleidomastoid muscle contraction level may lead to inaccurate results, which could mislead clinical diagnosis and intervention strategies. By ensuring sternocleidomastoid muscle contraction level is uniformly controlled bilaterally, practitioners can diminish both intra- and inter-individual variability in amplitude, thereby improving the sensitivity of disease diagnosis [54].

As surface EMG is the algebraic sum of trains of action potentials generated by active motor units as detected by electrodes placed on the skin [55], either the bulk of the SCM muscles or the distance from the surface electrodes to the SCM muscle may affect the p13-n23 amplitude. Due to the relatively thin subcutaneous fat layer in infants, larger p13-n23 amplitudes are often observed [56]. Consequently, cVEMP amplitudes in children are significantly larger than those in adults, which is why the amplitude of cVEMP has received less attention. However, the decrement of stimulus intensity will provide some more information compared to very higher intensity.

### Interaural asymmetry ratio (IAR)

The interpretation of IAR, especially in infants, should be approached with caution due to potential variation in testing condition, participant cooperation, and procedural accuracy. Longitudinal monitoring is crucial for the verification of unilateral vestibular dysfunctions. Disparity in reported normal ranges for these measurements underscore the imperative for additional multicenter research to refine and standardize these values, enhancing the diagnostic precision and reliability of cVEMP testing in clinic via sensitivity and speciality.

### cVEMP threshold

Threshold analysis in cVEMP is a critical component for assessing inner ear function, particularly in diagnosing conditions associated with “third window” syndromes such as semicircular canal dehiscence and large vestibular aqueduct syndrome [18, 57]. These conditions are characterized by markedly lower response threshold, often observed between 60 and 70 dB nHL [18, 57]. However, due to the need for rapid test completion and the challenges in securing cooperation with infants, threshold testing is typically not included in initial screening procedure.

### Determination of abnormal result

The evaluation should not be solely based on the presence or absence of the cVEMP waveform, but also on the results of its parameters. The 95% confidence intervals for the p13 and n23 latencies, amplitudes and IAR of cVEMP across different age groups in normal control population can be used as the normal reference range. A waveform is considered as abnormal if it is absent, or if the parameter falls outside the normal range. Any such discrepancy is considered an indication of an abnormal waveform.

The variability observed in the cVEMP response parameters can be attributed to a variety of influencing factors, making it imperative for each center to develop and adhere to its own set of age-specific normative values. The establishment of such reference standards within the various centers can help to generate more standardized data by which the clinical applicability and interpretive accuracy of cVEMP test results can be significantly improve.

### Conclusion of consensus

In summary, this consensus discusses the importance and feasibility of early VIS, specifically recommending cVEMP test at 3 months of age to optimize early detection and intervention strategies for vestibular dysfunctions in infants. It also highlights the superiority of BCV-cVEMP over ACS-cVEMP in this demographic, due to its ability to bypass conductive conditions in the external canal and middle ear, ensuring a more reliable assessment of vestibular function. The consensus also calls for the establishment of age-specific normative cVEMP parameters to enhance the precision of screening results.

For those showing abnormalities in initial screening, a subsequent diagnostic examination at 6 months of age is advised. This consensus addresses the importance of clinically assessing primitive developmental reflexes and motor function in infants to screen the vestibular system in addition. Moreover, it would draw more attention to the significant correlation between hearing loss and vestibular dysfunction, especially in infants at higher risk such as severe to profound SNHL, inner ear malformations, cCMV infections, post- cochlear implantation, and genetic syndrome, etc.

Advantages of cVEMP in VIS include its objectivity, non-invasiveness, and feasibility in very young infants. It specifically assesses saccular function and the inferior vestibular nerve pathway. However, limitations include dependency on adequate sternocleidomastoid muscle activation, technical training requirements, and its restriction to otolith function without evaluation of semicircular canals. In cases where VEMP responses are absent or inconclusive, or when broader vestibular assessment is clinically indicated, additional tests such as vHIT, RCT and Clinical motor developmental assessments should be considered based on the child’s age and cooperation [58].

This consensus advocates for the integration of vestibular screening within a comprehensive diagnostic framework that includes etiological diagnosis. This holistic approach aims to guide the development and implementation of VIS programs, ensuring that infants with vestibular dysfunction receive timely interventions, thereby improving their developmental outcomes and quality of life.

## References

[1] Erbek S, Gokmen Z, Ozkiraz S, Erbek SS, Tarcan A, Ozluoglu LN (2009) Vestibular evoked myogenic potentials in preterm infants. Audiol Neurootol 14:1–6. 10.1159/000148204 18663293 10.1159/000148204

[2] Young YH (2015) Assessment of functional development of the otolithic system in growing children: a review. Int J Pediatr Otorhinolaryngol 79:435–442. 10.1016/j.ijporl.2015.01.015 25650143 10.1016/j.ijporl.2015.01.015

[3] Cushing SL, Papsin BC (2019) Special considerations for the pediatric patient. Adv Otorhinolaryngol 82:134–142. 10.1159/000490282 30947211 10.1159/000490282

[4] Martens S, Dhooge I, Dhondt C, Leyssens L, Sucaet M, Vanaudenaerde S, Rombaut L, Maes L (2019) Vestibular infant screening - Flanders: the implementation of a standard vestibular screening protocol for hearing-impaired children in Flanders. Int J Pediatr Otorhinolaryngol 120:196–201. 10.1016/j.ijporl.2019.02.033 30849604 10.1016/j.ijporl.2019.02.033

[5] Shen J, Wang L, Ma X, Chen Z, Chen J, Wang X, He K, Wang W, Sun J, Zhang Q, Shen M, Chen X, Zhang Q, Kaga K, Duan M, Yang J, Jin Y (2022) Cervical vestibular evoked myogenic potentials in 3-month-old infants: comparative characteristics and feasibility for infant vestibular screening. Front Neurol 13:992392. 10.3389/fneur.2022.992392 36247765 10.3389/fneur.2022.992392 PMC9557108

[6] Ma X, Shen J, Sun J, Wang L, Wang W, He K, Chen X, Zhang Q, Jin Y, Gao D, Duan M, Yang J, Chen J, He J (2023) P300 event-related potential predicts cognitive dysfunction in patients with vestibular disorders. Biomedicines. 10.3390/biomedicines11092365 37760807 10.3390/biomedicines11092365 PMC10525252

[7] Wiener-Vacher SR, Hamilton DA, Wiener SI (2013) Vestibular activity and cognitive development in children: perspectives. Front Integr Neurosci 7:92. 10.3389/fnint.2013.00092 24376403 10.3389/fnint.2013.00092 PMC3858645

[8] Martens S, Dhooge I, Dhondt C, Vanaudenaerde S, Sucaet M, Rombaut L, Maes L (2023) Pediatric vestibular assessment: clinical framework. Ear Hear 44:423–436. 10.1097/AUD.0000000000001303 36534710 10.1097/AUD.0000000000001303

[9] Martens S, Maes L, Dhondt C, Vanaudenaerde S, Sucaet M, De Leenheer E, Van Hoecke H, Van Hecke R, Rombaut L, Dhooge I (2023) Vestibular infant screening-flanders: what is the most appropriate vestibular screening tool in hearing-impaired children? Ear Hear 44:385–398. 10.1097/AUD.0000000000001290 36534644 10.1097/AUD.0000000000001290

[10] Gedik-Soyuyuce O, Gence-Gumus Z, Ozdilek A, Ada M, Korkut N (2021) Vestibular disorders in children: a retrospective analysis of vestibular function test findings. Int J Pediatr Otorhinolaryngol 146:110751. 10.1016/j.ijporl.2021.110751 33964674 10.1016/j.ijporl.2021.110751

[11] Wiener-Vacher SR, Campi M, Boizeau P, Thai-Van H (2023) Cervical vestibular evoked myogenic potentials in healthy children: normative values for bone and air conduction. Front Neurol 14:1157975. 10.3389/fneur.2023.1157975 37143993 10.3389/fneur.2023.1157975 PMC10152971

[12] Inoue A, Iwasaki S, Ushio M, Chihara Y, Fujimoto C, Egami N, Yamasoba T (2013) Effect of vestibular dysfunction on the development of gross motor function in children with profound hearing loss. Audiol Neurootol 18:143–151. 10.1159/000346344 23392310 10.1159/000346344

[13] Maes L, De Kegel A, Van Waelvelde H, Dhooge I (2014) Rotatory and collic vestibular evoked myogenic potential testing in normal-hearing and hearing-impaired children. Ear Hear 35:e21–32. 10.1097/AUD.0b013e3182a6ca91 24556969 10.1097/AUD.0b013e3182a6ca91

[14] Verrecchia L, Galle Barrett K, Karltorp E (2020) The feasibility, validity and reliability of a child friendly vestibular assessment in infants and children candidates to cochlear implant. Int J Pediatr Otorhinolaryngol 135:110093. 10.1016/j.ijporl.2020.110093 32422368 10.1016/j.ijporl.2020.110093

[15] Hazen M, Cushing SL (2020) Implications of concurrent vestibular dysfunction in pediatric hearing loss. Curr Otorhinolaryngol Rep 8:267–275. 10.1007/s40136-020-00298-3

[16] Apeksha K, Singh S, Rathnamala M, Varalakshmi S, Preethu DJ, Kavya V, Sowndarya DS, Arpitha S, Milana K, Navya S, Thejasvi MA (2021) Balance assessment of children with sensorineural hearing loss. Indian J Otolaryngol Head Neck Surg 73:12–17. 10.1007/s12070-020-01797-x 33643879 10.1007/s12070-020-01797-x PMC7881998

[17] Janky KL, Thomas M, Patterson J, Givens D (2022) Using functional outcomes to predict vestibular loss in children. Otol Neurotol 43(3):352–358. 10.1097/MAO.0000000000003433 34802017 10.1097/MAO.0000000000003433 PMC8837677

[18] Shen M, Xue S, Wei X, Chen B, Kong Y, Li Y (2024) Characteristics of vestibular-evoked myogenic potentials in children with vestibular malformation and severe sensorineural hearing loss. Int J Pediatr Otorhinolaryngol 176:111781. 10.1016/j.ijporl.2023.111781 38006708 10.1016/j.ijporl.2023.111781

[19] Verrecchia L, Karpeta N, Westin M, Johansson A, Aldenklint S, Brantberg K, Duan M (2019) Methodological aspects of testing vestibular evoked myogenic potentials in infants at universal hearing screening program. Sci Rep 9:17225. 10.1038/s41598-019-53143-z 31754248 10.1038/s41598-019-53143-z PMC6872559

[20] Martens S, Dhooge I, Dhondt C, Vanaudenaerde S, Sucaet M, Rombaut L, Boudewyns A, Desloovere C, Janssens de Varebeke S, Vinck AS, Vanspauwen R, Verschueren D, Foulon I, Staelens C, Van den Broeck K, De Valck C, Deggouj N, Lemkens N, Haverbeke L, De Bock M, Oz O, Declau F, Devroede B, Verhoye C, Maes L (2020) Vestibular infant screening (vis)-flanders: results after 1.5 years of vestibular screening in hearing-impaired children. Sci Rep 10:21011. 10.1038/s41598-020-78049-z 33273502 10.1038/s41598-020-78049-z PMC7713061

[21] Dhondt C, Dhooge I, Maes L (2019) Vestibular assessment in the pediatric population. Laryngoscope 129:490–493. 10.1002/lary.27255 30394531 10.1002/lary.27255

[22] Sheykholeslami K, Megerian CA, Arnold JE, Kaga K (2005) Vestibular-evoked myogenic potentials in infancy and early childhood. Laryngoscope 115:1440–1444. 10.1097/01.mlg.0000167976.58724.22 16094120 10.1097/01.mlg.0000167976.58724.22

[23] Chen CN, Wang SJ, Wang CT, Hsieh WS, Young YH (2007) Vestibular evoked myogenic potentials in newborns. Audiol Neurootol 12:59–63. 10.1159/000097248 17119334 10.1159/000097248

[24] Erbek S, Erbek SS, Gokmen Z, Ozkiraz S, Tarcan A, Ozluoglu LN (2007) Clinical application of vestibular evoked myogenic potentials in healthy newborns. Int J Pediatr Otorhinolaryngol 71(8):1181–1185. 10.1016/j.ijporl.2007.04.007 17537524 10.1016/j.ijporl.2007.04.007

[25] Greenwalt NL, Patterson JN, Rodriguez AI, Fitzpatrick D, Gordon KR, Janky KL (2021) Bone conduction vibration vestibular evoked myogenic potential (vemp) testing: reliability in children, adolescents, and young adults. Ear Hear 42:355–363. 10.1097/AUD.0000000000000925 32701728 10.1097/AUD.0000000000000925 PMC7855257

[26] Fife TD, Tusa RJ, Furman JM, Zee DS, Frohman E, Baloh RW, Hain T, Goebel J, Demer J, Eviatar L (2000) Assessment: vestibular testing techniques in adults and children: report of the therapeutics and technology assessment subcommittee of the American academy of neurology. Neurology 55:1431–1441. 10.1212/wnl.55.10.1431 11094095 10.1212/wnl.55.10.1431

[27] Duarte DSB, Cabral AML, Britto D (2022) Vestibular assessment in children aged zero to twelve years: an integrative review. Braz J Otorhinolaryngol 88(Suppl 3):S212–S224. 10.1016/j.bjorl.2022.09.006 36347786 10.1016/j.bjorl.2022.09.006 PMC9761112

[28] De Kegel A, Maes L, Baetens T, Dhooge I, Van Waelvelde H (2012) The influence of a vestibular dysfunction on the motor development of hearing-impaired children. Laryngoscope 122:2837–2843. 10.1002/lary.23529 22990988 10.1002/lary.23529

[29] Cushing SL, Papsin BC, Rutka JA, James AL, Gordon KA (2008) Evidence of vestibular and balance dysfunction in children with profound sensorineural hearing loss using cochlear implants. Laryngoscope 118:1814–1823. 10.1097/MLG.0b013e31817fadfa 18758383 10.1097/MLG.0b013e31817fadfa

[30] Verbecque E, Marijnissen T, De Belder N, Van Rompaey V, Boudewyns A, Van de Heyning P, Vereeck L, Hallemans A (2017) Vestibular (dys)function in children with sensorineural hearing loss: A systematic review. Int J Audiol 56:361–381. 10.1080/14992027.2017.1281444 28264605 10.1080/14992027.2017.1281444

[31] Martens S, Dhooge I, Dhondt C, Vanaudenaerde S, Sucaet M, Van Hoecke H, De Leenheer E, Rombaut L, Boudewyns A, Desloovere C, Vinck AS, de Varebeke SJ, Verschueren D, Verstreken M, Foulon I, Staelens C, De Valck C, Calcoen R, Lemkens N, Oz O, De Bock M, Haverbeke L, Verhoye C, Declau F, Devroede B, Forton G, Deggouj N, Maes L (2022) Three years of vestibular infant screening in infants with sensorineural hearing loss. Pediatrics. 10.1542/peds.2021-055340 35698886 10.1542/peds.2021-055340

[32] Maes L, Dhooge I, D’Haenens W, Bockstael A, Keppler H, Philips B, Swinnen F, Vinck BM (2010) The effect of age on the sinusoidal harmonic acceleration test, pseudorandom rotation test, velocity step test, caloric test, and vestibular-evoked myogenic potential test. Ear Hear 31:84–94. 10.1097/AUD.0b013e3181b9640e 19779351 10.1097/AUD.0b013e3181b9640e

[33] Tribukait A, Bergenius J, Brantberg K (1996) The subjective visual horizontal for different body tilts in the roll plane: characterization of normal subjects. Brain Res Bull 40:375–381. 10.1016/0361-9230(96)00130-x 8886362 10.1016/0361-9230(96)00130-x

[34] Dhondt C, Maes L, Van Acker E, Martens S, Vanaudenaerde S, Rombaut L, De Cuyper E, Van Hoecke H, De Leenheer E, Dhooge I (2023) Vestibular follow-up program for congenital cytomegalovirus based on 6 years of longitudinal data collection. Ear Hear 44:1354–1366. 10.1097/AUD.0000000000001377 37122081 10.1097/AUD.0000000000001377

[35] Shears A, Yan G, Mortimer H, Cross E, Sapuan S, Kadambari S, Luck S, Heath PT, Walter S, Fidler KJ (2022) Vestibular and balance dysfunction in children with congenital CMV: a systematic review. Arch Dis Child Fetal Neonatal Ed 107:630–636. 10.1136/archdischild-2021-323380 35545420 10.1136/archdischild-2021-323380 PMC9606507

[36] Ersahan AA, Deger HM, Durgut M, Ozturk M, Mutlu F (2023) Long term effects of cochlear implant surgery on vestibular system in pediatric population. Auris Nasus Larynx 51:337–342. 10.1016/j.anl.2023.11.007 38071175 10.1016/j.anl.2023.11.007

[37] He L, Gong SS (2023) [impact of cochlear implant on vestibular function and the intervention strategies]. Zhonghua Er Bi Yan Hou Tou Jing Wai Ke Za Zhi 58:75–79. 10.3760/cma.j.cn115330-20221029-00639 36603872 10.3760/cma.j.cn115330-20221029-00639

[38] Pecuch A, Gieysztor E, Telenga M, Wolanska E, Kowal M, Paprocka-Borowicz M (2020) Primitive reflex activity in relation to the sensory profile in healthy preschool children. Int J Environ Res Public Health 17. 10.3390/ijerph17218210 10.3390/ijerph17218210 PMC7664452 33172138

[39] Awad R, Oropeza J, Uhler KM (2019) Meeting the joint committee on infant hearing standards in a large metropolitan children’s hospital: barriers and next steps. Am J Audiol 28:251–259. 10.1044/2019_AJA-18-0001 31084570 10.1044/2019_AJA-18-0001 PMC6802868

[40] Joint Committee on Infant H, American Academy of A, American Academy of P, American Speech-Language-Hearing A, Directors of S, Hearing Programs in State H, Welfare A (2000) Year 2000 position statement: Principles and guidelines for early hearing detection and intervention programs. Joint committee on infant hearing, american academy of audiology, american academy of pediatrics, american speech-language-hearing association, and directors of speech and hearing programs in state health and welfare agencies. Pediatrics 106:798–817. 10.1542/peds.106.4.798 10.1542/peds.106.4.798 11015525

[41] Farinetti A, Raji A, Wu H, Wanna B, Vincent C (2018) International consensus (ICON) on audiological assessment of hearing loss in children. Eur Ann Otorhinolaryngol Head Neck Dis 135:S41–S48. 10.1016/j.anorl.2017.12.008 29366866 10.1016/j.anorl.2017.12.008

[42] Papathanasiou ES, Murofushi T, Akin FW, Colebatch JG (2014) International guidelines for the clinical application of cervical vestibular evoked myogenic potentials: an expert consensus report. Clin Neurophysiol 125:658–666. 10.1016/j.clinph.2013.11.042 24513390 10.1016/j.clinph.2013.11.042

[43] Zhou G, Dargie J, Dornan B, Whittemore K (2014) Clinical uses of cervical vestibular-evoked myogenic potential testing in pediatric patients. Medicine 93:e37. 10.1097/MD.0000000000000037 25068952 10.1097/MD.0000000000000037 PMC4602421

[44] Thomas MLA, Fitzpatrick D, McCreery R, Janky KL (2017) Big stimulus, little ears: safety in administering vestibular-evoked myogenic potentials in children. J Am Acad Audiol 28:395–403. 10.3766/jaaa.15097 28534730 10.3766/jaaa.15097 PMC5443117

[45] Rodriguez AI, Thomas MLA, Fitzpatrick D, Janky KL (2018) Effects of high sound exposure during air-conducted vestibular evoked myogenic potential testing in children and young adults. Ear Hear 39:269–277. 10.1097/AUD.0000000000000484 29466264 10.1097/AUD.0000000000000484 PMC5826614

[46] Nguyen KD, Welgampola MS, Carey JP (2010) Test-retest reliability and age-related characteristics of the ocular and cervical vestibular evoked myogenic potential tests. Otol Neurotol 31:793–802. 10.1097/MAO.0b013e3181e3d60e 20517167 10.1097/MAO.0b013e3181e3d60e PMC2913294

[47] Curthoys IS, Kim J, McPhedran SK, Camp AJ (2006) Bone conducted vibration selectively activates irregular primary otolithic vestibular neurons in the Guinea pig. Exp Brain Res 175:256–267. 10.1007/s00221-006-0544-1 16761136 10.1007/s00221-006-0544-1

[48] Curthoys IS, Vulovic V, Burgess AM, Cornell ED, Mezey LE, Macdougall HG, Manzari L, McGarvie LA (2011) The basis for using bone-conducted vibration or air-conducted sound to test otolithic function. Ann N Y Acad Sci 1233:231–241. 10.1111/j.1749-6632.2011.06147.x 21950999 10.1111/j.1749-6632.2011.06147.x

[49] McCaslin DL, Jacobson GP, Hatton K, Fowler AP, DeLong AP (2013) The effects of amplitude normalization and EMG targets on Cvemp interaural amplitude asymmetry. Ear Hear 34(4):482–490. 10.1097/AUD.0b013e31827ad792 23361360 10.1097/AUD.0b013e31827ad792

[50] Kelsch TA, Schaefer LA, Esquivel CR (2006) Vestibular evoked myogenic potentials in young children: test parameters and normative data. Laryngoscope 116:895–900. 10.1097/01.mlg.0000214664.97049.3e 16735887 10.1097/01.mlg.0000214664.97049.3e

[51] Wu CH, Young YH, Murofushi T (1999) Tone burst-evoked myogenic potentials in human neck flexor and extensor. Acta Otolaryngol 119:741–744. 10.1080/00016489950180351 10687928 10.1080/00016489950180351

[52] Wang SJ, Yeh TH, Chang CH, Young YH (2008) Consistent latencies of vestibular evoked myogenic potentials. Ear Hear 29:923–929. 10.1097/AUD.0b013e3181853019 18685495 10.1097/AUD.0b013e3181853019

[53] Janky KL, Rodriguez AI (2018) Quantitative vestibular function testing in the pediatric population. Semin Hear 39:257–274. 10.1055/s-0038-1666817 30038454 10.1055/s-0038-1666817 PMC6054588

[54] Lee KJ, Kim MS, Son EJ, Lim HJ, Bang JH, Kang JG (2008) The usefulness of rectified vemp. Clin Exp Otorhinolaryngol 1(3):143–147. 10.3342/ceo.2008.1.3.143 19434246 10.3342/ceo.2008.1.3.143 PMC2671751

[55] Keenan KG, Farina D, Merletti R, Enoka RM (2006) Influence of motor unit properties on the size of the simulated evoked surface EMG potential. Exp Brain Res 169:37–49. 10.1007/s00221-005-0126-7 16273406 10.1007/s00221-005-0126-7

[56] Farina D, Arendt-Nielsen L, Merletti R, Graven-Nielsen T (2002) Assessment of single motor unit conduction velocity during sustained contractions of the tibialis anterior muscle with advanced spike triggered averaging. J Neurosci Methods 115:1–12. 10.1016/s0165-0270(01)00510-6 11897359 10.1016/s0165-0270(01)00510-6

[57] Zalewski CK, Chien WW, King KA, Muskett JA, Baron RE, Butman JA, Griffith AJ, Brewer CC (2015) Vestibular dysfunction in patients with enlarged vestibular aqueduct. Otolaryngol Head Neck Surg 153:257–262. 10.1177/0194599815585098 25968061 10.1177/0194599815585098 PMC8010515

[58] Coudert A, Brodsky JR, Dhooge I, Boudewyns A, Chiao A, Cushing S, Dasgupta S, Espinosa-Sanchez JM, Gurberg J, Ionescu E, Jenks CM, Lavender V, Maudoux A, R OR, Pagarkar W, Parodi M, Saniasiaya J, Tjernstrom F, Waissbluth S, Widdershoven JCC, Simon F (2025) International pediatric otolaryngology group (ipog) consensus on vestibular testing in children. 10.1002/lary.32261 10.1002/lary.32261 PMC12645366 40371981

